# Hybrid Deep Learning Based on a Heterogeneous Network Profile for Functional Annotations of *Plasmodium falciparum* Genes

**DOI:** 10.3390/ijms221810019

**Published:** 2021-09-16

**Authors:** Apichat Suratanee, Kitiporn Plaimas

**Affiliations:** 1 Department of Mathematics, Faculty of Applied Science, King Mongkut’s University of Technology North Bangkok, Bangkok 10800, Thailand; apichat.s@sci.kmutnb.ac.th; 2 Intelligent and Nonlinear Dynamic Innovations Research Center, Science and Technology Research Institute, King Mongkut’s University of Technology North Bangkok, Bangkok 10800, Thailand; 3 Advanced Virtual and Intelligent Computing (AVIC) Center, Department of Mathematics and Computer Science, Faculty of Science, Chulalongkorn University, Bangkok 10330, Thailand; 4 Omics Science and Bioinformatics Center, Faculty of Science, Chulalongkorn University, Bangkok 10330, Thailand

**Keywords:** heterogeneous network, hybrid deep learning, functional annotations, protein network profiles

## Abstract

Functional annotation of unknown function genes reveals unidentified functions that can enhance our understanding of complex genome communications. A common approach for inferring gene function involves the ortholog-based method. However, genetic data alone are often not enough to provide information for function annotation. Thus, integrating other sources of data can potentially increase the possibility of retrieving annotations. Network-based methods are efficient techniques for exploring interactions among genes and can be used for functional inference. In this study, we present an analysis framework for inferring the functions of *Plasmodium falciparum* genes based on connection profiles in a heterogeneous network between human and *Plasmodium falciparum* proteins. These profiles were fed into a hybrid deep learning algorithm to predict the orthologs of unknown function genes. The results show high performance of the model’s predictions, with an AUC of 0.89. One hundred and twenty-one predicted pairs with high prediction scores were selected for inferring the functions using statistical enrichment analysis. Using this method, *PF3D7_1248700* and *PF3D7_0401800* were found to be involved with muscle contraction and striated muscle tissue development, while *PF3D7_1303800* and *PF3D7_1201000* were found to be related to protein dephosphorylation. In conclusion, combining a heterogeneous network and a hybrid deep learning technique can allow us to identify unknown gene functions of malaria parasites. This approach is generalized and can be applied to other diseases that enhance the field of biomedical science.

## 1. Introduction

Identification of gene functions is a fundamental application for comparative genomic studies. Ortholog inference is a famous method for transferring functional annotations from one organism to another. Orthologous genes are used to demonstrate evolutionary relationships between different species, and it has been shown that orthologous genes often reveal significant functional similarities [1,2,3,4]. Identifying gene functions leads to a better understanding of the complexity of a genome. Malaria is a critical disease caused by *Plasmodium* parasites. The global technical strategy (GTS) for malaria from 2016 to 2030 aims to reduce the case incidence and mortality rates of malaria by at least 90% by 2030 from the 2015 baseline, as reported by the World Health Organization’s (WHO) malaria report in 2020. However, drug resistance has emerged as an issue, particularly in southeast Asia [5,6], which carries the potential to cause further spread. This worsening antimalarial resistance threatens plans for malaria treatment, control, and elimination. Among all *Plasmodium* species infecting humans, *Plasmodium falciparum* (*P. falciparum*) is the most common malarial parasite and the most likely to result in severe infections. It is fatal if not hastily treated. The first-line treatment for *P. falciparum* malaria is artemisinin in combination with a partner drug. *P. falciparum* parasites resistant to first-line therapies are found across Southeast Asia, particularly in the Greater Mekong Subregion (GMS) [7,8]. Moreover, we have a limited understanding of the immune mechanisms for malaria protection [9], and several *Plasmodium* genes remain functionally unannotated [10,11].

A common approach to functional inference for an annotated gene is to find orthologs. Many orthologous genes often retain similar biological functions preserved across species [4,12,13,14]. Despite the availability of *Plasmodium* genomes for more than a decade, there still remains many *Plasmodium* genes with unknown functions [10,15]. Genetic data alone are often not enough to provide information for functional annotations. Instead of using only genetic data, therefore, other information, such as associations and interactions between proteins, can be used to identify proteins in large-scale multiple connection views, which assist in inferring the functions of uncharacterized proteins [16,17], associations of proteins and drugs [18,19], and associations among diseases [20]. This knowledge potentially helps to reveal novel therapeutic approaches to treating malaria. A heterogeneous network—that is, a network connecting two or more different types of networks—has been applied in several studies [16,21,22]. Suratanee et al. [16] used a network propagation algorithm on a heterogeneous network to find associations between *Plasmodium vivax* and human proteins. To draw information from the network, topological features could be extracted. Liu et al. [21] extracted topological features from a heterogeneous network of drugs and diseases and used a deep neural network (DNN) to predict new drug–disease associations. A deep neural network was also used to predict protein–protein interactions from common protein descriptors [23]. Machine learning algorithms integrating network topology features of a heterogeneous network have been successfully used to identify parasite and human proteins [22].

One of the more famous machine learning algorithms is a technique known as deep learning. This technique is capable of extracting features directly from data and managing large-scale and high-dimension data. Therefore, deep learning methods have become unprecedentedly famous in several studies of biomedical applications, including learning protein sequences for protein contact prediction [24], protein structure prediction [25], and chemistry and drug design [26,27,28]. In particular, convolution neural networks (CNNs) have been successfully used to solve problems in computational biology. These can map spatial patterns from protein sequences and generate more complex features amenable to predicting protein structures [29,30]. Another famous deep learning method is the recurrent neural network (RNN). An RNN’s architecture contains loops wherein the output of a neuron is fed back to itself. The neural states that result from this looping enable the network to hold memory from the previous state. Therefore, RNNs obtain the present and recent past input sources and combine them to produce the current internal state. The final state will summarize the whole input sequence [30]. RNNs have been successfully used to solve many sequence-based problems, and they are suitable for predicting a protein structure based on protein sequences. However, RNNs can easily suffer from gradient vanishing or gradient explosion problems in which the error decreases or increases exponentially during training [31]. To alleviate these problems, a special RNN, known as long short-term memory (LSTM) [32], has been widely used. LSTM enables long-range dependencies by introducing an intermediate storage step within the memory cell that is controlled by gates. The gates can add or remove information to the cell state [33]. Bidirectional LSTM has been used for protein secondary structure prediction [34,35]. In addition, LSTM and CNN have been fused to predict eight classes of the secondary structure of proteins [36,37]. These techniques usually deal with long sequence data.

To infer functions across organisms using network analysis methods, a network with a high completion rate for interactions and protein functions is required. Under this assumption, the human protein network was selected for inferring functions. With many human proteins and *P. falciparum* proteins, we could obtain a broad view of the complex relationships within a network. Thus, in this study, we utilized a hybrid deep learning method by combining a CNN and LSTM to learn a high-dimensional network profile from the constructed heterogeneous network of human and *P. falciparum* proteins to infer gene functions. Ortholog information between human and *P. falciparum* genes was applied for deep learning. With the network profile and ortholog information between human and *P. falciparum* genes fed to the hybrid deep learning system, the prediction of human genes associated with *P. falciparum* genes was performed. Then, gene functions were inferred from the predicted human genes using statistical analysis. Finally, a list of functions annotated as being related to unknown function genes was compiled.

## 2. Results

### 2.1. Performance Evaluation of the Hybrid Deep Learning Method with Heterogeneous Network Profiles

Network connection profiles of all human and *Plasmodium* gene pairs were extracted from the heterogeneous network. These profiles were used for a binary classification to distinguish between known and unknown orthologs. However, the dimensions of the data were very large because all connections of a human protein corresponding to a human gene in a gene pair to all proteins in the human network and also connections of a *P. falciparum* protein corresponding to a *P. falciparum* gene in the gene pair to all proteins in the *P. falciparum* network were integrated. In addition, all connections of a protein corresponding to a gene pair to all proteins across the network of another organism were also integrated into the profiles. In total, 28,782 profiles were extracted for a given gene pair. To handle this large number of features, the convolutional neural network was employed to extract the latent features from these network connection profiles. In addition, we enhanced the performance of the classification process by introducing LSTM followed by CNN. A hybrid deep learning structure was constructed. Importantly, to avoid any biases in our performance evaluations, any connections between *Plasmodium* and human proteins found in the test set were excluded from the heterogeneous network during the training processes. This means that information about the protein pairs in the test set is not contained in the features of the training set in each experiment.

Five-fold cross-validations were performed to measure performance. For each fold, the training data were divided by 20% for validation. Several hyperparameters were optimized. The performances measured from the test set were obtained. The ranges of hyperparameter values are shown in Supplementary Table S1. Finally, we yielded the area under the receiver operating characteristics curve (AUC) with a standard deviation of 0.8787 ± 0.005 (Mean ± SD) and the area under precision recall curve (AUCPR) of 0.8724 ± 0.005. Selecting the cut-off showing the highest accuracy, we obtained ACC, REC, PREC, and F1 of 0.7997 ± 0.005, 0.8149 ± 0.007, 0.7910 ± 0.009, and 0.8027 ± 0.005, respectively.

### 2.2. Refining Heterogeneous Network Profiles for Classification

**Figure 1:**
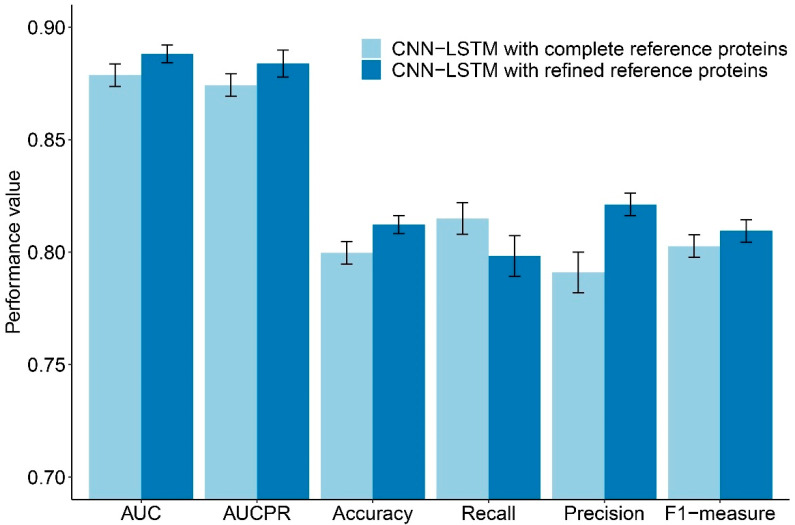
Average performances of CNN-LSTM with the complete reference proteins compared to CNN-LSTM with the refined reference proteins. The error bar indicates the standard deviation. The average AUC, AUCPR, Accuracy, Precision, and F1-measure of CNN-LSTM with the refined reference proteins are greater than the average performances of CNN-LSTM with the complete reference proteins.

With high-dimensional data, sparse features could be found. We attempted to reduce the number of features by selecting only relevant ones. Instead of using all proteins in both the *P. falciparum* and human networks for consideration, we selected only proteins found in the positive protein pairs. With this restriction, we obtained a set of reference proteins with 1694 *P. falciparum* proteins and 4032 human proteins. To obtain proteins that impacted the network structures and also related to the selected set, we sought high-degree proteins in the network that were enriched in the set of reference proteins. The thresholding method was performed by gradually decreasing the degree-cutoff, starting from the maximum degree of nodes in the network. The proteins that have a higher degree than the cutoff were selected and examined if they were enriched in the set of reference proteins. The enrichment test was done by a one-sided Fisher’s exact test with the false discovery rate (FDR) correction. We selected the highest optimal cutoff at which the selected proteins were enriched, with a *p*-value of < 0.0001 in the reference proteins. Then, the selected enriched proteins were integrated into the reference proteins. With this protein selection, we obtained two more *P. falciparum* proteins and 169 human proteins. Finally, we obtained 1696 *P. falciparum* proteins and 4201 human proteins to be our reference proteins. Therefore, the dimensions of our data with modified heterogeneous network features became 11,794 features, which is a 41% reduction from the original set. Classification using hybrid deep learning was performed with this modified set of features and yielded improved AUC, AUCPR, ACC, PREC, and F1 of 0.8881 ± 0.004, 0.8838 ± 0.006, 0.8122 ± 0.004, 0.8212 ± 0.005, and 0.8095 ± 0.005, respectively. Only the REC of the performance value of the CNN-LSTM with refined reference proteins was inferior to the value of the CNN-LSTM with complete reference proteins. The compared performances of the CNN-LSTM with complete reference proteins and the CNN-LSTM with refined reference proteins are shown in Figure 1.

In addition, we investigated the computational time when we performed the classifications with complete reference proteins from the heterogeneous network features compared to the computational time when we performed the classification with the refined heterogeneous features. The processing times of the hybrid model with the complete and refined reference proteins after running the five-times five-fold cross-validations in each experiment were analyzed. We found that the processing time of the hybrid model with the refined reference proteins was 2.9 times faster than that of the hybrid model with the complete reference proteins. The model with the refined reference proteins spent an average of 61,153 s (approximately 17 h) per experiment, while the model with the complete reference proteins spent an average of 178,589 s (approximately 49 h) per experiment. Therefore, the hybrid model with the refined reference proteins could improve the learning times.

### 2.3. Impact of the Reference Proteins on the Learning Process

From the results of the previous section, we found that reference proteins were important for the performance of classifications. Therefore, we performed experiments to investigate the impact of reference proteins. We considered proteins that were important for the network structures. The top proteins ranked by topological degree were selected to be the reference proteins. Accordingly, we performed the experiments by ranking proteins in the *P. falciparum* network and human network separately and selecting the top 10%, 20%, 30%, …, 90% of proteins from the ranked lists to be reference proteins. Each experiment was performed using the same procedures as the previous experiments. The results showed that the performance results of these experiments were inferior to the results from the previous sections. All results are shown in Supplementary Table S2. Moreover, we further attempted to reduce the number of reference proteins. With this consideration in mind, we reduced the set of proteins *D* using a single value decomposition (SVD) method and selected the reference proteins to be equal to the number of reference proteins from our refined features. The results showed very low performance for the classification, with an average AUC of 0.5358 and a standard deviation of 0.016. The results from our experiments demonstrated that our refined features still showed optimal results. Therefore, we used 1696 *P. falciparum* proteins and 4201 human proteins as our reference proteins for the final models.

### 2.4. Comparison to Other Prediction Methods

**Figure 2:**
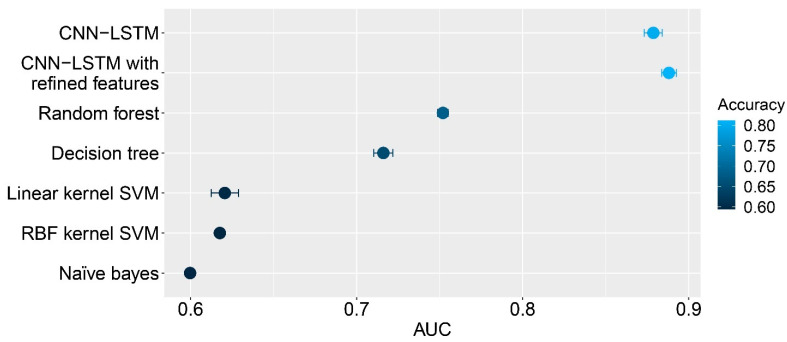
Performances of all classifiers, namely, CNN-LSTM, CNN-LSTM with refined features, random forest, decision tree, linear kernel SVM, RBF kernel SVM, and Naïve Bayes classifier. Each dot indicates the mean area under the receiver operating characteristic (ROC) curve of a classifier. The error bar indicates the standard deviation. Dot color reflects accuracy, as indicated in the legend.

We also ran other classification algorithms and compared their results to the performance of the hybrid deep learning method. Five different classifiers, consisting of random forest, decision tree, linear kernel support vector machine, radial basis function kernel support vector machine, and Naïve Bayes classifiers, were performed using the extracted features from various convolution kernels. Using the optimal kernel size of 1000, strides of 250 and 200 kernels were applied. In addition, 1D max pooling with a pool size of 20 and strides of five were set. The Glorot Uniform initializer [38] was used to initialize the kernel weights. Five experiments were performed to obtain the results. All hyperparameters of these classifiers were optimized. The results showed that the hybrid algorithms outperformed all standard methods. Among these four standard classifiers, the random forest yielded the best performance, with an average AUC of 0.7520 and a standard deviation of 0.003. The decision tree, linear kernel support vector machine, radial basis kernel support vector machine, and Naïve Bayes yielded an average AUC and standard deviation of 0.7161 ± 0.006, 0.6206 ± 0.008, 0.6176 ± 0.002, and 0.5997 ± 0.001, respectively. The respective performances of these methods are shown in Figure 2, and the respective performances of these classifiers are shown in Table 1.

As expected, the hybrid method demonstrated higher performance than the other methods. This is because the hybrid method combines the efficiency of both CNN and LSTM. The CNN comprised learned kernels in the convolution layer. The weight values in the kernels were learnable during the training phase. In addition, the CNN applied the same set of local convolutional filters used in one part of the input data across other parts of the input data. This process brings the advantage of avoiding overfitting problems. The pooling layer in the CNN helps to achieve translational invariance that the CNN is able to predict orthologs. If the connections among proteins and their neighboring are change slightly, it would not affect the values of most of the pooled outputs. The outputs from the CNN were fed to LSTM, which is capable of handling long-term dependencies with fusing ability and mapping to static classes of outputs.

### 2.5. Functional Annotations for Unknown Function Genes

**Figure 3:**
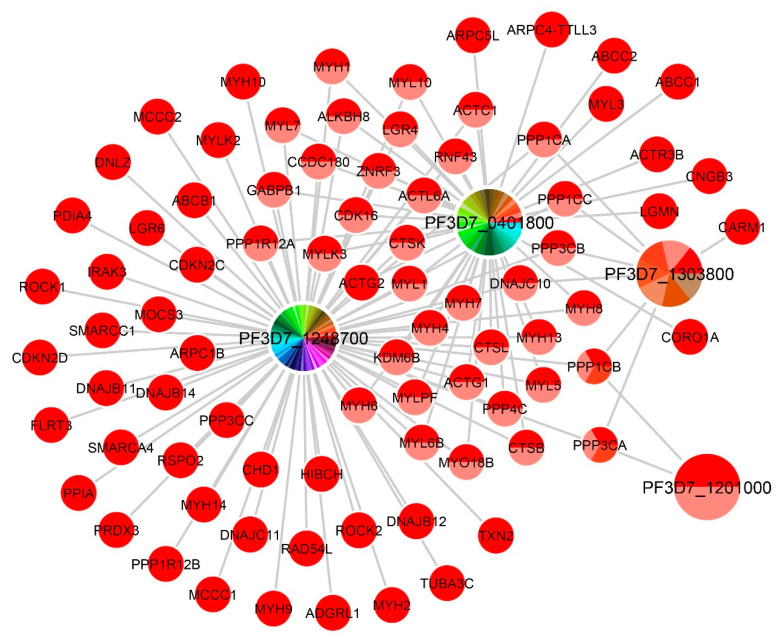
The network of predicted orthologous human genes of four unknown function *P. falciparum* genes, namely, *PF3D7_1248700*, *PF3D7_0401800*, *PF3D7_1303800*, and *PF3D7_1201000*. The pie chart at the node shows the number of neighboring nodes, corresponding to the number of slices. The colors in a node that shows different slices were rendered using the modulated rainbow color scheme. A node with a large number of colored slides indicates a large number of its neighboring nodes.

Hybrid deep learning was successfully used to predict orthologous genes between humans and *P. falciparum*. It is of great interest to further investigate the gene functions of *P. falciparum* genes. Functional inference was performed by integrating the predicted pairs of *P. falciparum* and human genes. Note that one *P. falciparum* gene could be predicted to be associated with more than one human gene. Only pairs with high prediction scores were investigated. We focused on the unknown function genes. Therefore, we sought unknown function *P. falciparum* genes from the PlasmoDB database and found 256 unknown function genes that were among our investigated genes in our network. We investigated the associations of these unknown genes with all the investigated human genes. A total of 3,081,458 pairs not found in our known orthologous pairs were obtained, and 270 pairs were found in the known set. These pairs with heterogeneous network profiles were predicted to be orthologous or not using 25 learned models. The average probability for each pair was calculated and assigned as a score for the pair. The scores of these two groups were investigated, and it was found that the pairs found in the known orthologous set showed significantly higher prediction scores than those of pairs found in the unknown orthologous set (*p*-value < 2.2 × 10^−17^). With this prediction score, we focused on pairs not found in the ortholog database. To obtain more precise prediction results, we selected a more stringent prediction score of more than 0.99. With this criterion, we obtained 121 pairs with four *P. falciparum* genes and 83 human genes. Then, we inferred the functions of these *P. falciparum* genes using functions of their orthologous human genes. Supplementary Table S3 shows all 121 pairs with their prediction scores, and the network of these 121 pairs is shown in Figure 3. From the figure, we can see that a *P. falciparum* gene can be associated with more than one human gene. In addition, these *P. falciparum* genes shared some common human genes. To demonstrate the degree of each gene, the nodes of the network were visualized in a pie chart indicating the number of neighboring nodes. To find the function of these unknown function *P. falciparum* genes, all human genes predicted to be orthologous to a *P. falciparum* gene were collected. The enrichment analysis of these human genes was performed on the gene ontology data to find the GO terms in biological processes that these genes overrepresented. With this analysis, we obtained the representative GO terms that were inferred to be the functions of the *P. falciparum* gene.

Table 2 shows the predicted gene functions of four unknown function *P. falciparum* genes. From the enrichment analysis, overrepresented GO terms in the human genes for each *P. falciparum* gene were obtained. The conserved protein *PF3D7_1248700* was predicted to be orthologous to 71 human proteins. We found five gene ontology terms that these human proteins enriched. These were muscle contraction, actin-myosin filament sliding, striated muscle tissue development, regulation of synaptic vesicle endocytosis, and entrainment of the circadian clock by photoperiod. Interestingly, classical symptoms of patients suffering from malaria infections include effects on skeletal muscle functions, such as muscle contractures, muscle aches, muscle pain, muscle fatigue, and muscle weakness [39,40]. For *PF3D7_0401800*, also known as the PfD80 *Plasmodium* exported protein (*PHISTb*), 38 human proteins were predicted to be orthologous. Five GO terms were enriched for the human proteins. Their enriched GO terms were related to the muscle system and were close to the GO terms for *PF3D7_1248700*, except that transepithelial transport and receptor catabolic process were different. For *PF3D7_1303800*, seven human proteins were predicted to be orthologous and four GO terms were enriched. Two of them were related to the circadian clock. The other two terms were protein dephosphorylation and regulation binding. The other *Plasmodium* exported protein (*PHISTb*), *PF3D7_1201000*, was predicted to be related to two human proteins consisting of the protein phosphatase 1 catalytic subunit beta (*PPP1CB*) and the protein phosphatase 3 catalytic subunit alpha (*PPP3CA*). These two proteins were not found to be enriched in any GO terms. Therefore, we focused on the GO terms of these two human proteins and used them as the gene functions for *PF3D7_1201000*. With this consideration, we found nine GO terms that protein dephosphorylation was included. Table 2 shows these three *P. falciparum* genes, and Supplementary Table S4 shows their enriched GO terms and all results of enrichment analysis in biological processes. The enrichment analysis results of the molecular functions and cellular components are presented in Supplementary Tables S5 and S6, respectively.

### 2.6. Investigating Relevant Functions from Neighboring Genes

**Figure 4:**
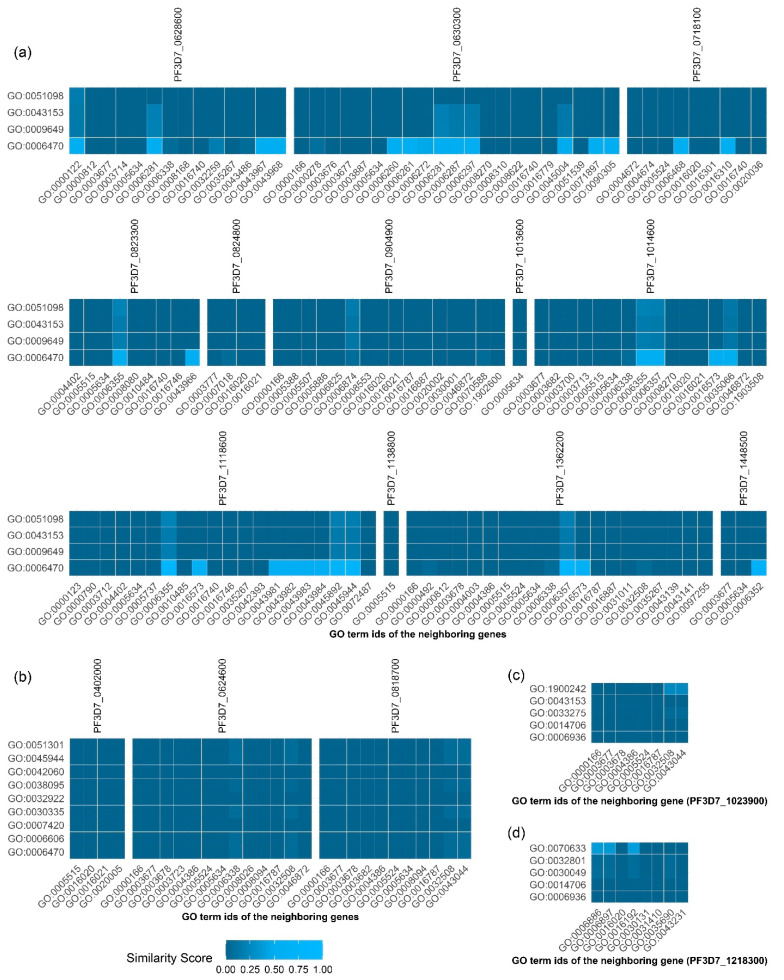
The similarities of the GO terms of four unknown function genes, including (**a**) *PF3D7_1303800*, (**b**) *PF3D7_1201000*, (**c**) *PF3D7_1248700*, and (**d**) *PF3D7_0401800*, plus the GO terms of their neighbors. The names of their neighboring genes are listed at the top of each block. The GO terms of the unknown function *P. falciparum* genes are listed on the y-axis, and the GO terms of their neighboring genes are listed on the x-axis. The heat map color reflects the similarity score, as indicated in the legend.

To investigate whether the inferred GO terms of a *P. falciparum* gene are similar or closely related to the GO terms of its neighboring gene, the neighboring genes of these four genes (*PF3D7_1248700*, *PF3D7_0401800*, *PF3D7_1303800*, and *PF3D7_1201000*) were observed in the *P. falciparum* network and their close or common functions were sought out. Only one neighbor gene of *PF3D7_1248700* was found: *PF3D7_1023900*. *PF3D7_0401800* also had one neighbor, *PF3D7_1218300*. For *PF3D7_1201000*, there were three neighboring genes: *PF3D7_0402000*, *PF3D7_0624600*, and *PF3D7_0818700*. For *PF3D7_1303800*, there were twelve neighboring genes: *PF3D7_0628600*, *PF3D7_0630300*, *PF3D7_0718100*, *PF3D7_0823300*, *PF3D7_0824800*, *PF3D7_0904900*, *PF3D7_1013600*, *PF3D7_1014600*, *PF3D7_1118600*, *PF3D7_1138800*, *PF3D7_1362200*, and *PF3D7_1448500*. GO term similarity was computed, and similarity scores ranging from 0 to 1 of *PF3D7_1303800*, *PF3D7_1201000*, *PF3D7_1248700*, and *PF3D7_0401800* are shown in Figure 4a–d, respectively.

For *PF3D7_1303800*, we found protein dephosphorylation (GO:0006470), which was close to other GO terms of *PF3D7_130800*’s neighbors. The highest similarity score, 0.88, was calculated between GO:0006470 and the regulation of transcription, DNA-templated by GO:0006355, of *PF3D7_1118600*. GO:0006470 also showed a high similarity score in relation to other GO terms of *PF3D7_1303800*’s neighbors with a similarity score of more than 0.7. These included GO:0006468, GO:0006357, GO:0045892, GO:0006281, GO0045944, GO:0016310, and GO:0000122. Therefore, protein dephosphorylation may be a primary function of *PF3D7_1303800*.

Interestingly, for *PF3D7_1201000*, we also found the function of protein dephosphorylation. However, the similarity scores of this function to the GO terms of *PF3D7_1201000*’s neighbor were quite low (less than 0.1). Similar to the GO:0006470, the GO terms for the *PF3D7_1201000* showed a low similarity score in relation to the GO term of its neighboring genes.

For *PF3D7_1248700*, we found the function of regulation of synaptic vesicle endocytosis (GO:1900242) to be closely related to DNA duplex unwinding (GO:0032508) and chromatin remodeling (GO:0043044) with a similarity score of 0.26. In addition, we also found a very low similarity (less than 0.1) of actin-myosin filament sliding (GO:0033275) with DNA duplex unwinding (GO:0032508) and chromatin remodeling (GO:0043044).

For *PF3D7_0401800*, transepithelial transport (GO:0070633) was related to vesicle-mediated transport (GO:0016192), intracellular protein transport (GO:0006886), and endocytosis (GO:0006897), with similarity scores of 0.4012, 0.3852, and 0.3702, respectively. Therefore, *PF3D7_0401800* may be related to transport processes.

**Figure 5:**
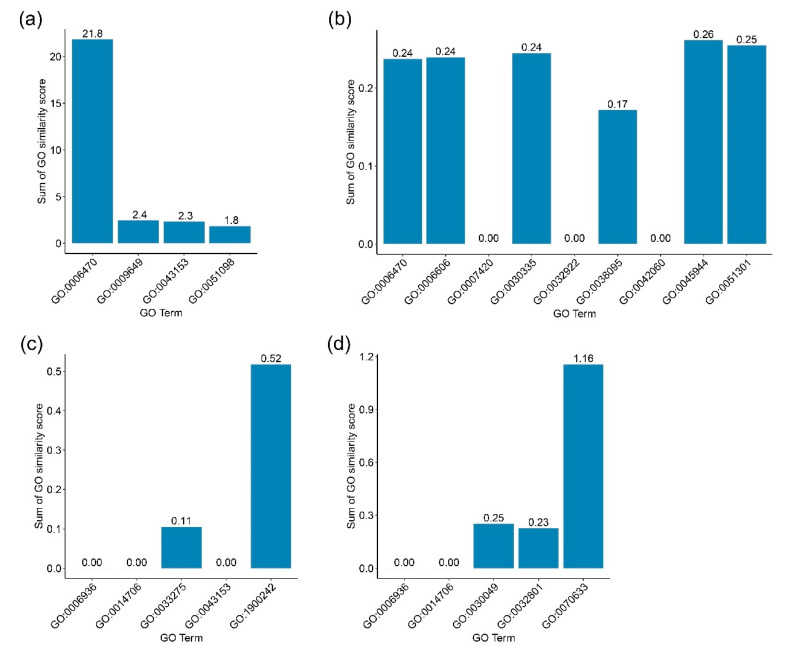
The sum of the GO similarity scores for the GO terms of the unknown function *P. falciparum* genes in relation to the GO terms of their neighboring genes. The bar plots (**a**–**d**) are the results for *PF3D7_1303800*, *PF3D7_1201000*, *PF3D7_1248700*, and *PF3D7_0401800*, respectively. For *PF3D7_1303800*, GO:0006470 is found with the highest sum of the similarity scores. For *PF3D7_1201000*, five GO terms with similarity scores around 0.25 are noticed. For *PF3D7_1248700*, GO:1900242 shows the highest score. For *PF3D7_0401800*, GO:0070633 shows the highest sum of similarity scores compared to the score of the other GO terms.

An unknown function gene might relate to more than one GO term. However, it is interesting to find the most relevant GO term for each investigated *P. falciparum* gene. Therefore, we calculated the sum of the GO similarity scores for each GO term and the GO terms of the gene’s neighbors. The results are shown in Figure 5. For *PF3D7_1303800*, we found that GO:0006470 presented the highest sum of the similarity scores. For *PF3D7_1201000*, five GO terms, consisting of GO:0006470, GO:0006606, GO:003035, GO:0045944, and GO:00051301, showed similar values for the scores. These were quite low. For *PF3D7_1248700*, we found GO:1900242 showed a sum of GO similarity score higher than the sum scores of other GO terms in the same group. For *PF3D7_0401800*, we found that GO:0070633 showed the highest sum scores compared to the score of other GO terms.

**Figure 6:**
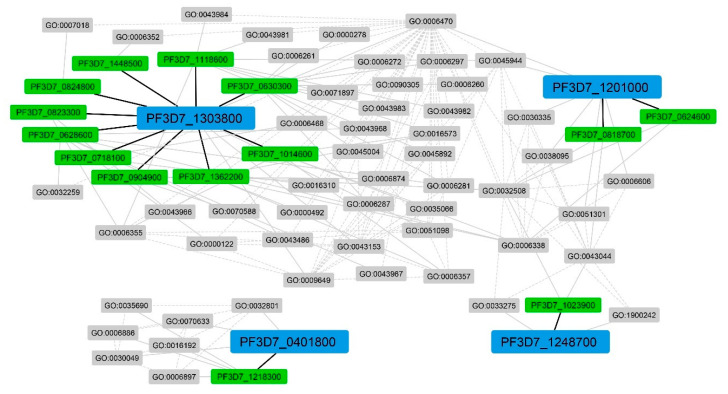
The network shows the connections among *PF3D7_1303800*, *PF3D7_1201000*, *PF3D7_1248700*, and *PF3D7_0401800*, their neighboring genes, and their corresponding GO terms. Notice that *PF3D7_1303800* has ten neighboring genes while the others have only one or two neighboring genes. Therefore, many GO terms can be found for *PF3D7_1303800*. There are some commonly shared GO terms among *PF3D7_1303800*, *PF3D7_1201000*, and *PF3D7_1248700*.

From the abovementioned, we found that protein dephosphorylation was highly related to other GO terms. To further investigate this term and observe other terms, we aggregated all investigated interactions and constructed a network, which is illustrated in Figure 6. Only neighboring genes with at least one of their GO terms showing a similar GO score of more than 0 compared to the GO terms of the unknown function genes were presented in the figure. From the network, we found that GO:0006470 showed a high value for degree centrality: 38. In addition, this term was directly and indirectly related to *PF3D7_1303800*, *PF3D7_1201000*, and *PF3D7_1248700*, except *PF3D7_0401800*, whose subnetwork was separated from others. Dephosphorylation processes are important for the regulation of signaling pathways, and protein dephosphorylation was remarkably effective for therapeutic strategies, especially in the regulation of *P. falciparum* [41].

## 3. Conclusions and Discussion

In this study, we proposed an analysis framework for predicting the functions of unknown function *P. falciparum* genes using the CNN-LSTM model. Network connection profiles were obtained from the constructed heterogeneous network, and these profiles were fed to the hybrid deep learning system to predict the orthologs between *P. falciparum* and human genes. The CNN-LSTM method was capable of handling a large number of heterogeneous network profiles. Our experiments have shown that our model can precisely predict orthologs with high performance and that it outperforms typical classifiers. In this work, the human network was used because it contains a high number of reliable interactions with a large number of annotated functions, and it is a host of *P. falciparum*. However, from the large number of interactions, we obtained a large set of features, which expended a lot of computer memory and computational time. In addition, network profiles with sparse features were usually found, especially in network features extracted from incomplete networks. Therefore, we refined the features to obtain a smaller set of features that used less memory and time but still had high classification performance.

From the deep learning algorithm, the orthologs were predicted based on the heterogeneous network features that integrated several pieces of information regarding interactions between gene products. The results show that a *P. falciparum* gene can be predicted to be related to more than one human gene. These results are useful because we were able to identify several functions from the predicted human genes using the enrichment test. Although we performed the clustering of the GO terms to obtain only the top-ranking GO term in each cluster that was a function for the *P. falciparum* gene, the GO terms with the second or third ranking with significant *p*-values also warrant further investigation. To obtain more relevant gene functions, we considered the functions of the neighboring *P. falciparum* genes and investigated whether they were closely related. Although the functions of the neighboring genes are not necessarily similar, they could be used as clues for inferring or filtering the related functions of the given *P. falciparum* gene. From our selected four unknown function *P. falciparum* genes, we found that some of them might be related to muscle functions and dephosphorylation processes important for *P. falciparum* regulation. Our functional annotation based on the heterogeneous network using a hybrid deep learning method is comprehensive and could be performed for other organisms.

## 4. Materials and Methods

### 4.1. Heterogeneous Network Construction and Ortholog Collection

To construct a reliable network, high-quality datasets need to be used. For the *P. falciparum* interaction network and the human interaction network, we employed interactions from the STRING database (version 11.0) (Copenhagen, Denmark; Heidelberg, Germany; Lausanne, Switzerland) [42]. Only confidence interactions with confidence values greater than or equal to 900 were collected. Accordingly, two interaction networks were reconstructed. The associations between *P. falciparum* and human proteins were functionally annotated based on orthologs that were carried out using EggNOG (version 5.0) (Heidelberg, Germany) [43], which is a widely used database providing comprehensive functional annotations of proteins. With this database, the ortholog associations between *P. falciparum* proteins and human proteins were retained. Finally, 12,038 human proteins and 2353 *P. falciparum* proteins were obtained. In total, 313,359 interactions between human and human, 36,050 interactions between *P. falciparum* and *P. falciparum*, and 11,024 interactions between human and *P. falciparum* proteins were obtained. Apart from the orthologous information from EggNOG and interaction information from STRING, we obtained functional annotation data of the *P. falciparum* gene from PlasmoDB [44] (accessed on 29 May 2021; Athens, Georgia, USA; Philadelphia, Pennsylvania, USA) for our analysis.

### 4.2. Heterogeneous Network Profile Extraction

To find the network profile describing the relationship of any two nodes in a heterogeneous network, complete connections between one node and the other nodes, either among the same or different types in the network, can be extracted. One piece of precious data that integrates information from all nodes of the network is feature extraction from the heterogeneous network. Sets of features have been successfully applied for the identification of drug–disease associations [21] and are known as heterogeneous network profiles. Thus, in this work, we also extracted these profiles from the heterogeneous network to find *Plasmodium* and human protein associations.

Considering m *Plasmodium* proteins and n human proteins in the heterogeneous network, we have expressed the following: $A_{\left(m\times m\right)}^{pp}$, representing a *Plasmodium*–*Plasmodium* protein interaction matrix; $A_{\left(n\times n\right)}^{hh}$, representing a human–human protein interaction matrix; and $A_{\left(m\times n\right)}^{ph}$, representing a *Plasmodium*–human interaction matrix. To extract a heterogeneous feature $F^{p_{i}h_{j}}$ for a pair of *Plasmodium* and human protein associations from the network, we constructed four vectors corresponding to $p_{i}$ and $h_{j}$, extracted from $A_{\left(m\times m\right)}^{pp}$, $A_{\left(n\times n\right)}^{hh}$, and $A_{\left(m\times n\right)}^{ph}$. Then, we concatenated these vectors to obtain the heterogeneous network feature for a pair.

The first vector, $a^{p_{i}P}=\left(a_{i1}^{pp},\;a_{i2}^{pp},\;a_{i3}^{pp},\;\ldots ,\;a_{im}^{pp}\right)=A^{pp}(p_{i},:)$, is a vector of protein $p_{i}$ to all *Plasmodium* proteins. The second, $a^{p_{i}H}=\left(a_{i1}^{ph},\;a_{i2}^{ph},\;a_{i3}^{ph},\;\ldots ,\;a_{in}^{ph}\right)=A^{ph}(p_{i},:)$, is a vector of protein $p_{i}$ to all human proteins. The third vector, $a^{Ph_{j}}={\left(a_{1j}^{ph},\;a_{2j}^{ph},\;a_{3j}^{ph},\;\ldots ,\;a_{mj}^{ph}\right)}^{T}=A^{ph}(:,h_{j})$, is a vector of protein $h_{j}$ to all *Plasmodium* proteins. Finally, $a^{h_{j}H}=\left(a_{j1}^{hh},\;a_{j2}^{hh},\;a_{j3}^{hh},\;\ldots ,\;a_{jn}^{hh}\right)=A^{hh}(h_{j},:)$ is a vector of protein $h_{j}$ to all human proteins. The heterogeneous network feature of a pair $\left(p_{i},\;h_{j}\right)$ is defined as $F^{p_{i}h_{j}}=a^{p_{i}P}\;⊕\;a^{p_{i}H}\;⊕\;a^{Ph_{j}}\;⊕\;a^{h_{j}H}$. The symbol ⊕ denotes concatenation of the feature vectors. This feature vector is generated for all possible pairs of $p_{i}$ and $h_{j}$ in the heterogeneous network.

### 4.3. Refining the Heterogeneous Network Profile Relative to the Entirety of Heterogeneous Network Information

The heterogeneous network profile containing the connection features for a pair $\left(p_{i},\;h_{j}\right)$ could be used to represent all network information between $p_{i}$ and the other nodes in the network and also between $h_{j}$ and the other nodes in the network. It could be used effectively for both small and moderated network models. However, for our large-scale analysis of a number of *Plasmodium* and human proteins in the network, a very large number of features for each protein pair would be generated. In addition, the obtained features could be a sparse vector because of the rare number of known protein interactions across the two species. To reduce redundant information, a refined set of features could be proposed. Instead of considering the interactions of a *Plasmodium* protein and a human one for a given protein pair to all proteins in the network, we focused on a smaller set of influenced proteins called reference proteins (D). The proteins in D were *P. falciparum* proteins and human proteins found in the set of known orthologous proteins. We used the denotation that $D_{P}$ is the set of *Plasmodium* proteins in D and $D_{H}$ is the set of human proteins in D, where $D=D_{P}\cup D_{H}$. Moreover, we realized there was some precious network topological information. Accordingly, we investigated the proteins that influence the network structures. With this consideration, we extracted high-degree proteins in both the *P. falciparum* and human networks that enriched in the set of $D_{P}$ and $D_{H}$, respectively, and included them in the reference proteins D. For the modified heterogeneous feature, the vectors $a^{p_{i}P}$, $a^{p_{i}H}$, $a^{Ph_{j}}$, and $a^{h_{j}H}$ could be adapted. We made the following definitions: $A^{pp*}=A^{pp}(:,D_{P})$, $A^{hh*}=A^{hh}(:,D_{H})$, $A^{ph*}=A^{ph}(:,\;D_{H})$, and $A^{p*h}=A^{ph}(D_{P},\;:)$. Therefore, the refined heterogeneous feature for a pair $\left(p_{i},\;h_{j}\right)$ is generated by

(1) $$\begin{array}{cc} & F_{refined}^{p_{i}h_{j}}=a^{p_{i}P*}⊕a^{p_{i}H*}⊕a^{P*h_{j}}⊕a^{h_{j}H*},\;\mathrm{where}\\ & a^{p_{i}P*}=\left(a_{i1}^{pp*},\;a_{i2}^{pp*},\;a_{i3}^{pp*},\;\ldots ,a_{iu}^{pp*}\right)=A^{pp*}(p_{i},:),\\ & a^{p_{i}H*}=\left(a_{i1}^{ph*},\;a_{i2}^{ph*},\;a_{i3}^{ph*},\;\ldots ,\;a_{iv}^{ph*}\right)=A^{ph*}(p_{i},:),\;\\ & a^{P*h_{j}}={\left(a_{1j}^{p*h},\;a_{2j}^{p*h},\;a_{3j}^{p*h},\;\ldots ,\;a_{uj}^{p*h}\right)}^{T}=A^{p*h}(:,h_{j}),\;\mathrm{and}\\ & a^{h_{j}H*}=\left(a_{j1}^{hh*},\;a_{j2}^{hh*},\;a_{j3}^{hh*},\;\ldots ,\;a_{jv}^{hh*}\right)=A^{hh*}(h_{j},:). \end{array}$$

Suppose that the number of *Plasmodium* and human proteins in D are u and v, respectively. Given this, the number of refined heterogeneous network features is equal to 2×(u+v). The refined heterogeneous network features were used in conjunction with a deep learning model to determine an association between *Plasmodium* and human proteins.

### 4.4. Hybrid Deep Learning Model Construction and Assessment

**Figure 7:**
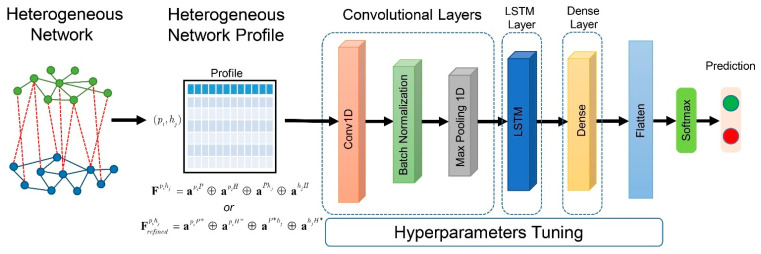
Overview of the analytical framework. Heterogeneous network of human and *P. falciparum* is constructed. Network profiles are extracted from the heterogeneous network. The network profiles are fed to the hybrid deep learning system to predict the orthologous genes between humans and *P. falciparum*.

Determining whether two proteins are orthologous or not is a binary classification problem. To perform the classification, we utilize a hybrid deep learning method that combines a CNN and LSTM. This is known as a convolutional long-short term memory (CNN-LSTM) network. The CNN-LSTM architecture uses CNN layers to extract information from input features and combine it with LSTMs to make predictions. Specifically, for the heterogeneous network feature, we used a 1D convolutional neural network on the CNN layer. The architecture and hyperparameters were optimized using the Adam algorithm [45], which is a stochastic gradient descent method based on adaptive estimation of first-order and second-order moments. The hyperparameters were tuned using a random search algorithm. The list of hyperparameters is shown in Supplementary Table S1. The architecture of the hybrid deep learning model is shown in Figure 7. These heterogeneous network profiles were fed as inputs to the CNN-LSTM model. The model started with a layer of 1D-CNN for extracting information from the heterogeneous features. The sigmoid function was used as the activation function at this stage. After the 1D-CNN layer was processed, batch normalization and the max pooling layer were applied. The output from the pooling layer was fed as an input to the LSTM layer. In the LSTM, a unit dropout technique was applied to the inputs to avoid an overfitting problem. A fully connected dense layer with a rectified linear activation function is applied to the LSTM output. The latent features from the dense layer were flattened. Then, a dense layer with a Softmax activation function was employed as the final layer.

To evaluate the performance of the model, 5-fold cross-validations were conducted. The data were randomly partitioned into five folds. For each fold, a single fold was retained as validation data for testing the model, and the remaining four folds were used for learning the model. Five repetitions of 5-fold cross-validations were performed. In each fold, the data were balanced in a one-to-one ratio by randomly under-sampling the majority class to train the algorithm. Overall, we performed 5-fold cross-validations for five experiments. To evaluate the predictive ability of the model, six measurements, including area under curve (AUC), area under precision recall curve (AUCPR), accuracy (ACC), precision (PREC), recall (REC), and the F1-measure (F1), were used. The ACC, PREC, REC, and F1 were calculated as follows:

(2) $$\begin{array}{cc} & \mathrm{ACC}=\frac{\left(TP+TN\right)}{\left(TP+FP+FN+TN\right)},\\ & \mathrm{PREC}\;=\;\frac{TP}{\left(TP+FP\right)},\\ & \mathrm{REC}=\frac{TP}{\left(TP+FN\right)},\\ & F1=\frac{2\times \mathrm{PREC}\times \mathrm{REC}}{\left(\mathrm{PREC}+\mathrm{REC}\right)}. \end{array}$$

*TP*, *TN*, *FP*, and *FN* are true positive, true negative, false positive, and false negative, respectively. The positive set is the set of known orthologous proteins, and the negative set consists of the undefined orthologous proteins. The high-performance models are the models that have high values for the above metrics.

### 4.5. Statistical Analysis for Functional Annotations

The results from the predictions consisted of pairs of *P. falciparum* and human proteins. To infer the functions of unknown function proteins of *P. falciparum*, we investigated the functions of human proteins predicted to be orthologous to *P. falciparum* proteins. Notice that one *P. falciparum* protein can be matched to more than one human protein. In this case, enrichment analysis could be employed to find the relevant functions of those human proteins. To this end, we employed an enrichment test using the ClusterProfilers [46] package in R (Guangzhou, China) to determine the gene ontology (GO) terms in biological processes overrepresented in the set of human proteins. A two-sided hypergeometric test with Benjamin–Hochberg corrections (*p*-value less than 0.01) was performed to find the significant GO terms. Similar GO terms were grouped based on their descriptions. The most representative of a given GO group was identified using the Revigo tool [47] (Zagreb, Croatia; Barcelona, Spain). To find the closeness between two GOs, a similarity measure of gene ontology from the GoSim package [48] in R (Heidelberg, Germany; Tuebingen, Germany) was employed. With this package, GO term similarity was measured using Lin’s pairwise similarity [49]. The network visualization was performed using Cytoscape version 3.8.2 [50].

## Figures and Tables

**Table 1:** The performance of CNN-LSTM with the refined reference proteins compared to the standard classifiers.

Method	AUC	AUCPR	ACC	REC	PREC	F1
CNN-LSTM with complete reference proteins	0.8787 ± 0.005	0.8724 ± 0.005	0.7997 ± 0.005	0.8149 ± 0.007	0.7910 ± 0.009	0.8027 ± 0.005
CNN-LSTM with refined reference proteins	0.8881 ± 0.004	0.8838 ± 0.006	0.8122 ± 0.004	0.7983 ± 0.009	0.8212 ± 0.005	0.8095 ± 0.005
Random forest	0.7520 ± 0.003	0.7534 ± 0.004	0.6836 ± 0.002	0.6641 ± 0.013	0.6912 ± 0.006	0.6773 ± 0.005
Decision Tree	0.7161 ± 0.006	0.7235 ± 0.008	0.6526 ± 0.002	0.5877 ± 0.043	0.6769 ± 0.019	0.6279 ± 0.015
Linear kernel SVM	0.6206 ± 0.008	0.6174 ± 0.008	0.5977 ± 0.009	0.4256 ± 0.047	0.6496 ± 0.009	0.5129 ± 0.032
RBF kernel SVM	0.6176 ± 0.002	0.6139 ± 0.001	0.5941 ± 0.001	0.4106 ± 0.024	0.6493 ± 0.010	0.5026 ± 0.015
Naïve Bayes classification	0.5997 ± 0.001	0.5999 ± 0.002	0.5948 ± 0.001	0.3908 ± 0.007	0.6603 ± 0.005	0.4910 ± 0.005

**Table 2:** The predicted gene functions for selected *P. falciparum* genes.

*P. falciparum* Gene Symbol	GO ID (Human)	GO Term (Human)	Corrected *p*-Value
*PF3D7_1248700*	GO:0006936	muscle contraction	4.16 × 10^−14^
	GO:0033275	actin-myosin filament sliding	5.80 × 10^−12^
	GO:0014706	striated muscle tissue development	1.00 × 10^−5^
	GO:1900242	regulation of synaptic vesicle endocytosis	0.00177
	GO:0043153	entrainment of circadian clock by photoperiod	0.00541
*PF3D7_0401800*	GO:0006936	muscle contraction	6.62 × 10^−14^
	GO:0030049	muscle filament sliding	1.33 × 10^−12^
	GO:0014706	striated muscle tissue development	2.56 × 10^−6^
	GO:0070633	transepithelial transport	0.00103
	GO:0032801	receptor catabolic process	0.00164
*PF3D7_1303800*	GO:0006470	protein dephosphorylation	1.17 × 10^−5^
	GO:0009649	entrainment of circadian clock	1.82 × 10^−5^
	GO:0043153	entrainment of circadian clock by photoperiod	1.82 × 10^−5^
	GO:0051098	regulation of binding	0.00234
*PF3D7_1201000*	GO:0006470	protein dephosphorylation	N/A
	GO:0006606	protein import into nucleus	
	GO:0007420	brain development	
	GO:0030335	positive regulation of cell migration	
	GO:0032922	circadian regulation of gene expression	
	GO:0038095	Fc-epsilon receptor signaling pathway	
	GO:0042060	wound healing	
	GO:0045944	positive regulation of transcription by RNA polymerase II	
	GO:0051301	cell division	

## Data Availability

Not applicable.

## Supplementary Materials

The following are available online at https://www.mdpi.com/article/10.3390/ijms221810019/s1.
